# Apatinib for recurrent/progressive glioblastoma multiforme: A salvage option

**DOI:** 10.3389/fphar.2022.969565

**Published:** 2022-08-17

**Authors:** Hong-Hong Zhang, Xiao-Jing Du, Mei-Ling Deng, Lie Zheng, Dun-Chen Yao, Zhi-Qiang Wang, Qun-Ying Yang, Shao-Xiong Wu

**Affiliations:** ^1^ Department of Radiation Oncology, Sun Yat-sen University Cancer Center, State Key Laboratory of Oncology in South China, Collaborative Innovation Center for Cancer Medicine, Guangzhou, China; ^2^ Department of Radiation Oncology, Xiang’an Hospital of Xiamen University, Cancer Research Center, School of Medicine, Xiamen University, Xiamen, China; ^3^ Department of Medical Imaging, Sun Yat-sen University Cancer Center, State Key Laboratory of Oncology in South China, Collaborative Innovation Center for Cancer Medicine, Guangzhou, China; ^4^ Department of Neurosurgery, Sun Yat-sen University Cancer Center, State Key Laboratory of Oncology in South China, Collaborative Innovation Center for Cancer Medicine, Guangzhou, China

**Keywords:** apatinib, recurrent/progressive glioblastoma multiforme, efficacy, safety, VEGFR

## Abstract

**Purpose:** The recurrent/progressive glioblastoma multiforme (GBM) carries a dismal prognosis and the definitive treatment strategy has not yet been established. This study aimed to assess the efficacy and safety of apatinib in recurrent/progressive GBM patients.

**Materials and methods:** The clinical data of 19 recurrent/progressive GBM patients who received apatinib treatment from November 2015 to December 2019 at Sun Yat-sen University Cancer Center were collected retrospectively in this study. Objective response rate (ORR), disease control rate (DCR), progression-free survival (PFS), overall survival (OS), and treatment-related adverse events (AEs) were reviewed and assessed.

**Results:** The overall ORR was 52.6%, and the DCR was 73.7%. Median PFS and OS were 5.1 and 10.4 months, respectively. The 6-month PFS and OS rates were 38.9% and 68.4%, respectively. The 12-month PFS and OS rates were 16.7% and 36.8%, respectively. The treatment-related toxicities were generally well-tolerated. The most common grade 3/4 AEs were hand-foot syndrome (36.8%) and hypertension (21.1%).

**Conclusion:** Our study showed that apatinib therapy provided a better salvaging option for recurrent/progressive GBM patients and the toxicity was manageable.

## Introduction

Glioblastoma multiforme (GBM) is a highly aggressive brain tumor. After conventional standard treatments, including surgery, chemotherapy, and radiotherapy, many patients eventually experience tumor recurrence or progression (Mckinnon et al., 2021). However, there is no recognized standard management for recurrent/progressive GBM and the prognosis is rather poor, with the median overall survival (OS) of only 2–9 months for recurrent GBM (Weller et al., 2013; Audureau et al., 2018; Weller and Le Rhun, 2020). Therefore, more effective therapeutic strategies are urgently needed for recurrent/progressive GBM.

With the development of molecular targeted therapy, various therapeutic drugs targeting epidermal growth factor receptor, angiogenesis, or programmed death-1 have been tried in the treatment of recurrent/progressive GBM. Among them, only the anti-angiogenesis agent bevacizumab, a humanized monoclonal antibody targeting vascular endothelial growth factor (VEGF), showed a promising capacity to prolong progression-free survival (PFS) in recurrent/progressive GBM patients, based on which it was approved for the treatment of relapsed GBM by Food and Drug Administration in 2009 (Vredenburgh et al., 2007; Friedman et al., 2009). Nevertheless, treatment failure is quite common as the emergence of drug resistance is inevitable.

Angiogenesis, an essential step in the tumor growth of malignant gliomas, is not only regulated by VEGF but also by vascular endothelial growth factor receptor (VEGFR) (Melincovici et al., 2018). Among all the VEGFR family proteins, VEGFR-2 is considered to be the most critical regulator of the angiogenesis process and a potential target for anti-cancer therapy (Shah et al., 2021). Apatinib, an oral small molecular tyrosine kinase inhibitor targeting VEGFR-2, has shown strong anti-cancer effect as well as the capacity of reversing multidrug resistance (MDR) induced by breast cancer resistance protein, MDR-associated protein 1, and MDR protein 1 (Mi et al., 2010; Tong et al., 2012; Yang et al., 2020). At present, apatinib has achieved promising efficacy in various types of solid tumors and has been approved by the National Medical Products Administration for the treatment of advanced gastric cancer or gastroesophageal adenocarcinoma and advanced hepatocellular carcinoma (Li et al., 2013; Scott et al., 2015; Li et al., 2016; Li et al., 2020).

Currently, only a few case reports, retrospective and prospective studies with small sample sizes indicated that apatinib might be effective in the treatment of recurrent glioma (Wang et al., 2017; Zhang et al., 2017; Ding et al., 2018; Wang et al., 2019; Ge et al., 2020; Hui et al., 2021; Zhu et al., 2022). Hence, we conducted this retrospective study to provide more clinical evidence of apatinib as a salvage therapy in recurrent/progressive GBM patients.

## Materials and methods

### Patients selection

The clinical data of patients diagnosed with recurrent/progressive GBM and treated with apatinib from November 2015 to December 2019 at Sun Yat-sen University Cancer Center were retrieved from patients’ medical history retrospectively. The inclusion criteria were as follows: histologically proven GBM as the primary tumor; histologically or radiologically diagnosed tumor recurrence or progression after conventional standard treatments including surgery, radiotherapy, and temozolomide; at least one measurable or assessable tumor lesion by magnetic resonance imaging (MRI); no serious heart, kidney or liver insufficiency; a signed consent form was provided by the patient before apatinib treatment. Pseudoprogression or radiation necrosis diagnosed by multidisciplinary team according to response assessment in neuro-oncology criteria (RANO) (Wen et al., 2010) was excluded from this study. This study was conducted with the approval of the Institutional Review Board of Sun Yat-sen University Cancer Center.

### Treatment

Apatinib (Jiangsu Hengrui Pharmaceuticals Co., Ltd., People’s Republic of China) was orally administered at a dose of 500 mg once daily until disease progression, death, or intolerable toxicity. Apatinib could be temporarily suspended, or reduced to 250 mg or increased to 750 mg once daily, or discontinued in a patient with severe adverse events (AEs). In the event of grade 3 or 4 AEs, a dose interruption for first occurrence were required until recovery to ≤ grade 2 and a dose reduction to 250 mg for recurrence. If the toxicity reoccurs after dose reduction, discontinue apatinib. If no AEs occurred for 14 days after starting apatinib, the dose could be increased to 750 mg.

### Efficacy and safety assessments

MRI scan was performed 1 month after apatinib treatment and every 2 months thereafter or when there were significant progression signs or other conditions that required evaluation of treatment effectiveness. Treatment responses were assessed according to RANO. Treatment responses included complete response (CR), partial response (PR), stable disease (SD), and progressive disease (PD). Objective response rate (ORR) referred to the incidence rate of CR plus PR. Disease control rate (DCR) referred to the proportion of patients who achieved CR, PR, and SD. PFS referred to the period from the beginning of apatinib treatment to disease progression or death. OS referred to the period from the beginning of apatinib treatment to death of any cause or last follow-up visit.

All AEs, from patients’ medical history, laboratory examination results, imaging reports, and telephone follow-up, were reviewed and evaluated according to the National Cancer Institute Common Terminology Criteria version 4.0. The AEs that might be related to apatinib were recorded as treatment-related AEs.

### Statistical analysis

All the data analyses were performed by Statistical Package for the Social Sciences, version 20.0 (SPSS, Chicago, IL, United States) and R, version 3.6.1 (http://www.r-project.org/). Kaplan-Meier method was used to evaluate survival and calculate survival rates, and the log-rank test was used for comparison.

## Results

### Patient characteristics

The data were collected on a total of 19 patients with recurrent/progressive GBM, whose tumor recurrence or progression was collectively discussed by physicians from radiation oncology, imaging and neurosurgery based on the patient’s imaging (MRI, functional MR or positron emission tomography) or pathological findings from re-excision/biopsy. Nine patients (47.4%) had new lesions outside of the radiation field, and 2 of them progressed within 3 months after radiotherapy. Ten patients (52.6%) had obvious tumor enlargement and persistent clinical deterioration attributable to tumor, which were considered as tumor recurrence or progression in the primary site by multidisciplinary team. The patients’ clinical characteristics were shown in Table 1. Eleven patients (57.9%) with karnofsky performance status (KPS) score < 80 were mainly due to neurological deficits before the initiation of apatinib. Apatinib was used as the first salvage therapy in 11 patients (57.9%) and second-line or above therapy in 8 patients (42.1%).

**TABLE 1:** Patient characteristics (*n* = 19).

Characteristics	N (%)
Sex	
Male	9 (47.4)
Female	10 (52.6)
Age	
Median (range), years	47 (19–63)
The extent of first surgery	
Total resection	9 (47.4)
Partial resection	10 (52.6)
IDH 1/2 status	
Mutated	0
Wild	14 (73.7)
Not done/unknown	5 (26.3)
MGMT status	
Methylated	8 (42.1)
Unmethylated	7 (36.8)
Not done/unknown	4 (21.1)
Re-surgery	
Yes	2 (10.5)
No	17 (89.5)
Re-irradiation	
Yes	2 (10.5)
No	17 (89.5)
Time from first diagnosis to apatinib use	
Median (range), months	14 (4.6–80.9)
≤ 12 months	7 (36.8)
> 12 months	12 (63.2)
Time from completion of chemoradiotherapy to last recurrence or progression	
Median (range), weeks	40.9 (4.1–139.9)
< 12 weeks	4 (21.1)
≥ 12 weeks	15 (78.9)
Number of recurrent/progressive lesions	
Single	12 (63.2)
Multiple	7 (36.8)
KPS score before apatinib	
≥ 80	8 (42.1)
< 80	11 (57.9)
Apatinib as the first salvage therapy	
Yes	11 (57.9)
No	8 (42.1)
Bevacizumab prior to apatinib	
Yes	2 (10.5)
No	17 (89.5)
Application of corticosteroid	
Yes	4 (21.1)
No	15 (78.9)

Abbreviations: IDH, isocitrate dehydrogenase; MGMT, O6-methylguanine-DNA methyltransferase; KPS, Karnofsky performance status.

### Efficacy

**FIGURE 1:**
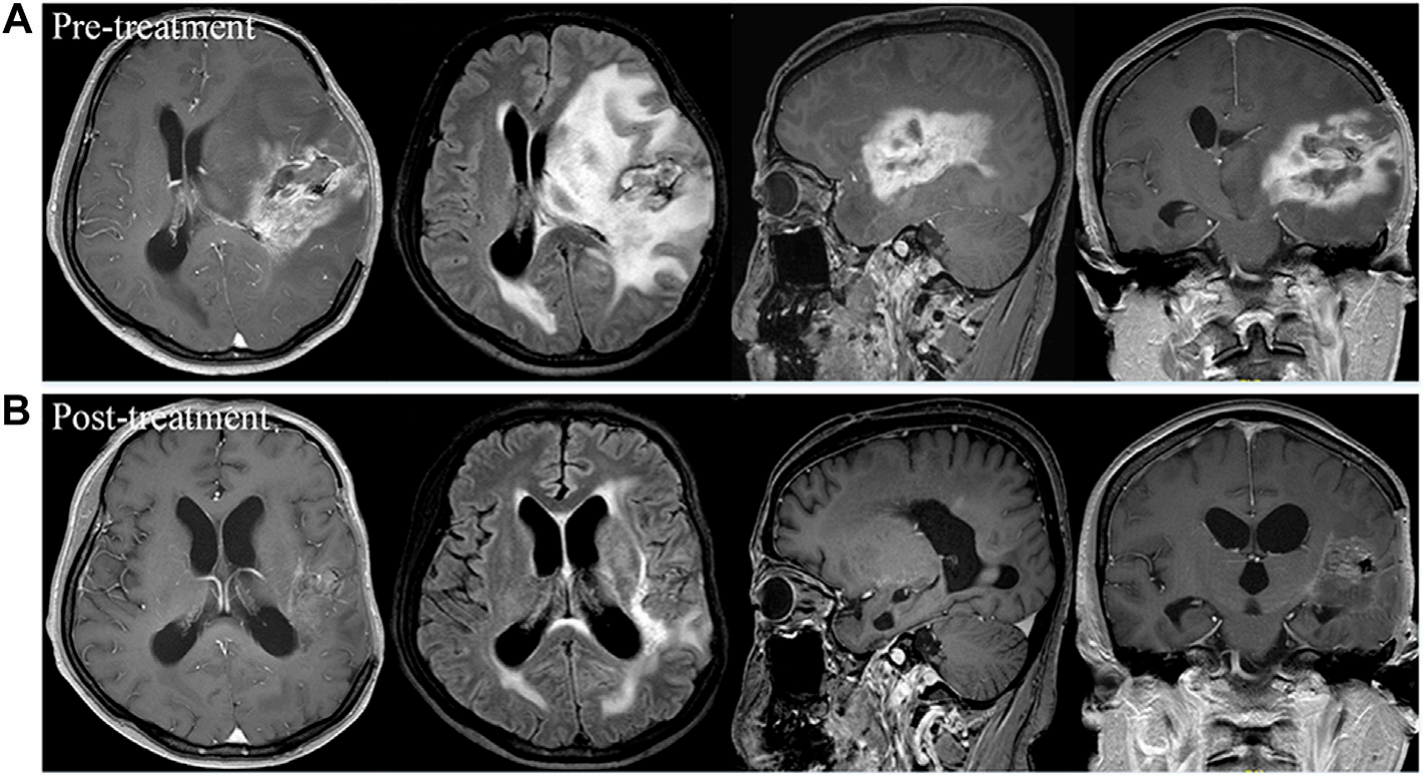
Brain scan of a patient with left frontotemporal glioblastoma multiforme who had relapsed from surgery, standard chemoradiotherapy regimen, and ten cycles of adjuvant temozolomide, and then had progressed after bevacizumab treatment. Comparison of imaging findings between pre-treatment **(A)** and post-treatment at 4 months of apatinib monotherapy **(B)** by contrast-enhanced MRI and MRI-Flair. The patient achieved partial response after treatment and had a progression-free survival time of 5.3 months.

**FIGURE 2:**
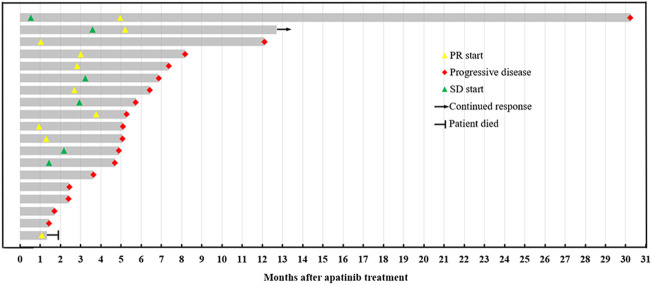
Swimmer plot of treatment responses.

Of the 19 patients, 10 (52.6%) patients had PR (Figure 1), 4 (21.1%) had SD, and 5 (19.2%) had PD (Figure 2). The overall ORR and DCR were 52.6% and 73.7%, respectively. For the 2 patients who failed to bevacizumab treatment before receiving apatinib, 1 patient achieved PR and 1 patient progressed.

**FIGURE 3:**
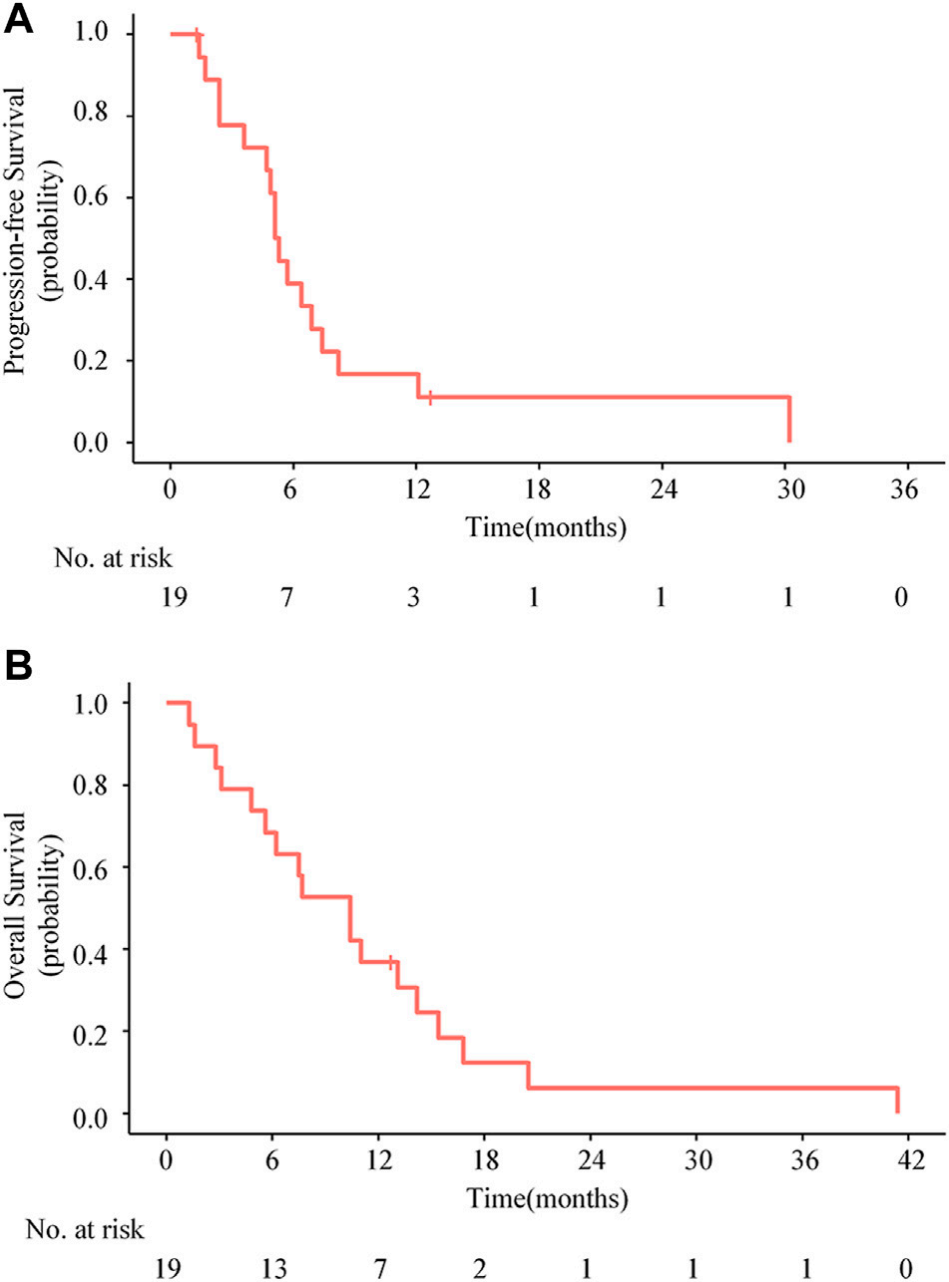
Kaplan-Meier estimate for progression-free survival **(A)** and overall survival **(B)** for all patients.

As of September 2020, the median follow-up time was 10.4 months (range 1.3–41.4 months). Disease progression occurred in 17 (89.5%) patients, and death occurred in 18 (94.7%) patients, including 1 patient who died from non-tumor-related cause. The median PFS was 5.1 months (95% CI, 4.5–5.7 months), with the estimated PFS rates of 38.9% (95% CI, 21.8%–69.4%) at 6 months and 16.7% (95% CI, 5.9%–46.8%) at 12 months, respectively (Figure 3A). The median OS was 10.4 months (95% CI, 6.3–14.5 months), with the estimated OS rates of 68.4% (95% CI, 50.4%–92.9%) at 6 months and 36.8% (95% CI, 20.4%–66.4%) at 12 months, respectively (Figure 3B).

Before the treatment of apatinib, 14 (73.7%) patients had poor clinical symptoms, including unilateral limb weakness (57.9%), cognitive dysfunction (42.1%), dizziness (36.8%), headache (36.8%), language impairment (31.6%) and vomiting (15.8%). After the administration of apatinib, 8 (57.1%) patients experienced the symptom relief. The median time to onset of relief was 18 days (range 7–40 days). Among those with pre-treatment KPS < 80, 45.5% (5/11) patients had an increase in KPS after 1 month of apatinib therapy, with the median KPS increasement of 20.

Four patients received 5–10 mg dexamethasone before apatinib to reduce symptoms of intracranial hypertension (eg, headache, vomiting), of which the dose of corticosteroid was reduced gradually after the initial of apatinib due to significant alleviation in symptoms and was completely discontinued on days 11–19 in all patients.

### Safety

The safety analysis was performed on all patients. The treatment-related toxicities were generally accepted. All AEs are summarized in Table 2. The most frequent AEs were hand-foot syndrome (HFS, 42.1%), hypertension (36.8%), decreased appetite (21.1%) and oral mucositis (21.1%). HFS was the most common grade 3 AE, with an incidence rate of 36.8% (7/19). No patient had grade 4 AEs. All AEs could be controlled by dose reduction, interruption or discontinuation of medication. Of all the 19 patients, 1 (5.3%) experienced a dose increase; 7 (36.8%) experienced a dose reduction; 4 (21.1%) discontinued the medication temporarily. No patient discontinued the medication permanently due to AEs.

**TABLE 2:** Adverse events.

Adverse events	Grade 1	Grade 2	Grade 3	Grade 4	All grade
	N (%)	N (%)	N (%)	N (%)	N (%)
Non-hematological events					
HFS	0	1 (5.3)	7 (36.8)	0	8 (42.1)
Hypertension	3 (15.8)	0	4 (21.1)	0	7 (36.8)
Decreased appetite	4 (21.1)	0	0	0	4 (21.1)
Oral mucositis	1 (5.3)	2 (10.5)	1 (5.3)	0	4 (21.1)
Proteinuria	1 (5.3)	0	2 (10.5)	0	3 (15.8)
Fatigue	3 (15.8)	0	0	0	3 (15.8)
Hemorrhage	0	2 (10.5)	0	0	2 (10.5)
Hoarseness	0	1 (5.3)	0	0	1 (5.3)
Diarrhea	0	0	1 (5.3)	0	1 (5.3)
Nauseous	1 (5.3)	0	0	0	1 (5.3)
Vomiting	1 (5.3)	0	0	0	1 (5.3)
Hematological events					
Leukopenia	1 (5.3)	1 (5.3)	2 (10.5)	0	4 (21.1)
ALT elevation	3 (15.8)	0	0	0	3 (15.8)
Total bilirubin elevation	3 (15.8)	0	0	0	3 (15.8)
Thrombocytopenia	1 (5.3)	1 (5.3)	0	0	2 (10.5)
AST elevation	1 (5.3)	0	0	0	1 (5.3)
Anemia	1 (5.3)	0	0	0	1 (5.3)

Abbreviations: HFS, hand-foot syndrome; ALT, Alanine aminotransferase; AST, Aspartate aminotransferase.

### Association of adverse events with clinical efficacy of apatinib

**FIGURE 4:**
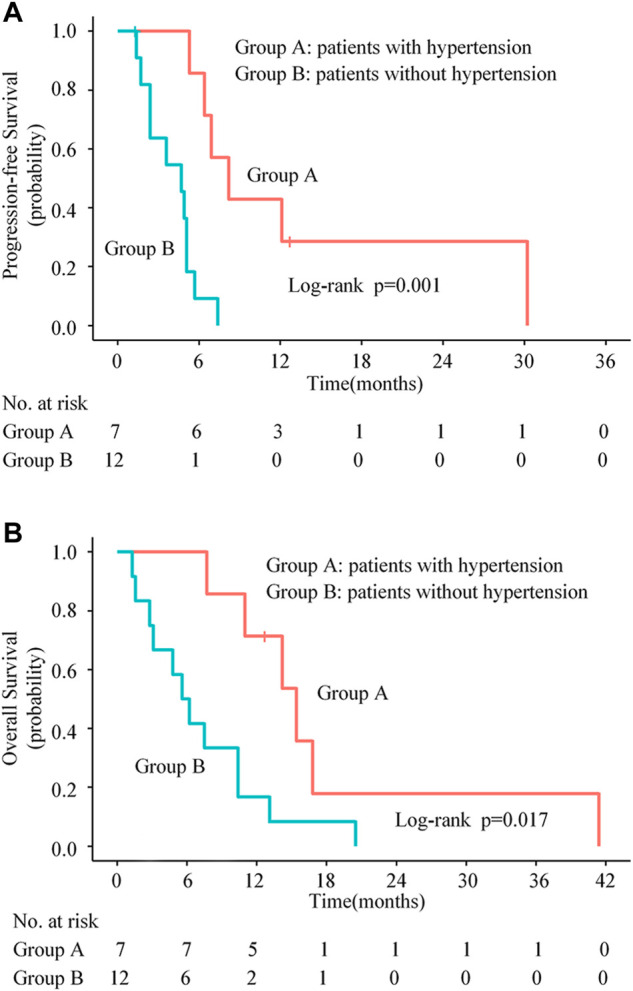
Kaplan-Meier progression-free survival curves **(A)** and overall survival curves **(B)** for patients stratified by experiencing hypertension or not.

Previous studies have reported that the emergence of specific AEs during anti-angiogenic therapy might be associated with better clinical outcomes (Ravaud and Schmidinger, 2013; Lee et al., 2016). In this retrospective study, we also found that patients with hypertension had significantly longer median PFS (8.2 months vs. 4.7 months, *p* = 0.001, Figure 4A) and OS (15.4 months vs. 5.6 months, *p* = 0.017, Figure 4B) compared with those without hypertension. Compared with those without HFS, patients with HFS had significantly longer median PFS (6.4 months vs. 3.6 months, *p* = 0.013) and a tendency of longer median OS (11.0 months vs. 5.6 months, *p* = 0.064).

## Discussion

The efficacy of apatinib in a variety of solid tumors (eg, gastric cancer, non-small cell lung cancer) has been confirmed in several clinical trials (Scott, 2018; Tian et al., 2021). In recurrent high-grade gliomas, preliminary evidence from a few exploratory trials has showed a promising clinical outcome with apatinib-based therapy, with the median PFS of 4–8.3 months, the median OS of 9–9.1 months and the ORR of 23.5%–55.0% (Wang et al., 2017; Wang et al., 2019; Hui et al., 2021). However, the application of apatinib monotherapy for recurrent/progressive GBM in clinical practice is rare reported, and only two patients with refractory recurrent malignant gliomas in our hospital were shared their treatment experience in the form of a case series (Zhang et al., 2017). To our knowledge, our study is the first study committed to investigating the efficacy and safety of apatinib monotherapy for patients with recurrent/progressive GBM in clinical practice.

In this retrospective study, the ORR, the median PFS and OS were 52.6%, 5.1 months (95% CI, 4.5–5.7 months) and 10.4 months (95% CI, 6.3–14.5 months), respectively, which seemed to be better than the data of apatinib plus temozolomide for recurrent GBM in an observational study reported by Ge et al. (2020), with the ORR, the median PFS and the median OS were 26.3%, 4.9 months (95% CI, 2.8–7 months) and 8.2 months (95% CI, 6.9–9.5 months), respectively. Differing from previous studies mentioned above focusing on apatinib-based combination therapy, all patients in our study were treated with apatinib monotherapy, further suggesting the effectiveness of single-agent apatinib against recurrent/progressive GBM.

At present, bevacizumab alone or combined with irinotecan is widely used as anti-angiogenic regimen for recurrent/progressive GBM treatment (Vredenburgh et al., 2007; Friedman et al., 2009; Zhang et al., 2012; Wick et al., 2017). The advantage of apatinib over bevacizumab is its convenient oral mode of administration, meaning that patients receiving apatinib do not require hospitalization and may improve their compliance and economic benefits. Also, the clinical outcomes of apatinib in our preliminary results were encouraging compared with those of bevacizumab. In a meta-analysis study regarding the treatment of recurrent GBM, the mean ORR of bevacizumab monotherapy was 33.9% and that of bevacizumab combined with irinotecan was 45.8%. The 6-month PFS were 38.8% and 48.3%, and the median OS were 8.63 months and 8.91 months for these two treatment regimens, respectively (Zhang et al., 2012). Our results in this study indicated that apatinib alone might be a promising therapeutic option for recurrent/progressive GBM. Furthermore, apatinib might still be effective even when the patients had already failed to bevacizumab. Of the 2 patients who progressed on bevacizumab, 1 had PR with apatinib in this study. Similar findings were also reported in another study conducted on patients with heavily treated metastatic colorectal cancer (Liang et al., 2018). Although the mechanism why apatinib remains effective after bevacizumab failure is unclear, it may be associated with the different location of the VEGF signaling pathway where bevacizumab acts on VEGF-A antigen on the tumor cell membrane, whereas apatinib acts on VEGFR-2 in tumor cells (Grothey and Galanis, 2009; Ahir et al., 2020). On the other hand, it may also be related to the fact that apatinib could reduce the formation of vasculogenic mimicry, which is considered to contribute to the development of resistance to bevacizumab (Yao et al., 2013; Ahir et al., 2020).

In line with the results in other case reports (Song et al., 2018), the symptoms of patients in our study were also quickly relived after apatinib treatment. We speculate that the therapeutic effect of apatinib is partially due to the rapid improvement of peritumoral brain edema by anti-angiogenic action in addition to the inherent anti-tumor effect. The destruction of the structure and function of blood-brain barrier is considered to be the pathological foundation of peritumoral brain edema (Song et al., 2018). Bevacizumab has been proven effective in dealing with tumor-associated brain edema and radiation-induced brain necrosis by blocking the VEGF/VEGFR signal transduction, repairing the abnormal blood vessels, and decreasing vascular permeability (Gonzalez et al., 2007; Sadraei et al., 2015; Shen et al., 2015). Recently, apatinib was also reported to be effective in treating refractory radiation-induced brain edema (Hu et al., 2017). In addition, of the 4 patients who administrated dexamethasone prior to apatinib to relieve brain edema in this study, all patients reduced the dose of corticosteroid quickly and ultimately discontinued as apatinib was administrated and the symptoms were significantly relieved, which also illustrates that apatinib has a significant anti-edema efficacy from another side.

In patients treated with apatinib, toxicities associated with traditional chemotherapy were rare, while other specific toxic effects, such as HFS and hypertension, were quite common (Peng et al., 2018). In this study, all toxicities were manageable, and the most frequent AEs of any grade and grade 3 were also HFS and hypertension. Additionally, we found that the patients with hypertension and HFS were associated with better prognoses. Liu et al. also observed the similar results in their study that the occurrence of proteinuria, HFS, or hypertension during the first cycle of apatinib treatment was a feasible biomarker predicting better anti-tumor effectiveness and longer OS in metastatic gastric cancer patients (Liu et al., 2017). The mechanisms underlying hypertension and HFS remain unclear. With regard to hypertension, previous studies has demonstrated that inhibition of VEGFR-2 can reduce the vascular density, thus leading to increased peripheral vascular resistance and eventually resulting in hypertension (Steeghs et al., 2006). In patients with metastatic renal cell cancer, van et al. found that VEGFR-2 blockade related capillary rarefaction was significantly correlated with prolonged PFS and OS (van der Veldt et al., 2010). Rini et al. had a hypothesis that the susceptibility of normal blood vessels to VEGF blockade, resulting in hypertension, was linked to the susceptibility of tumor vessels to VEGF blockade, leading to a stronger antiangiogenic effect (Rini et al., 2011). This may be the underlying biological basis of hypertension as a biomarker of VEGF blockade. With regard to HFS, several studies reported that VEGF pathway inhibition might be an essential factor affecting the pathophysiology and pathogenesis of HFS. The HFS might be attributable to the reduction of skin reconstruction after restriction of vessels (Azad et al., 2009; Fischer et al., 2013). Thus, HFS can serve as a biomarker of the efficacy of VEGF pathway inhibition because it may partly reflect the inherent host biology as a result of VEGF blockade.

Despite the interesting findings of our study, the limitations of the small sample size, lack of a control group and retrospective nature could not be ignored. We have to admit that some potential biases, such as self-selection bias and confounding bias, may affect the results. Currently, we are carrying out a multi-center phase 2 trial with a larger sample size to provide more reliable evidence for the application of apatinib monotherapy in treating recurrent/progressive GBM.

## Conclusion

Apatinib might be a better salvaging therapeutic option for recurrent/progressive GBM patients with an acceptable safety profile. This encouraging result requires to be further confirmed in more clinical trials.

## Data Availability

The raw data supporting the conclusions of this article will be made available by the authors, without undue reservation.
